# Medical Students Volunteering during COVID-19 Pandemic: Synopsis of Some Student-led Initiatives in Nepal

**DOI:** 10.31729/jnma.6703

**Published:** 2021-08-31

**Authors:** Pragyan Basnet, Anjali Joshi

**Affiliations:** 1 Patan Academy of Health Sciences, Lagankhel, Lalitpur, Nepal; 2 Kathmandu Medical College and Teaching Hospital, Sinamangal, Kathmandu, Nepal

**Keywords:** *coronavirus disease 2019*, *pandemic*, *personal protective equipment*, *plasma*, *telemedicine*

## Abstract

The efforts shown by healthcare professionals, security personnel and the genera! public in fighting Coronavirus Disease 2019 pandemic is highly appreciable. Medical students are future healthcare professionals and have the opportunity to volunteer and help their seniors fight Coronavirus Disease 2019 pandemic. In Nepal, we as medical students are contributing by raising awareness about Coronavirus Disease 2019 along with some innovative initiatives which are Project Personal Protective Equipment, Project Telemedicine and the "Donate Plasma, Help Defeat COVID-19" Campaign. Since we were able to support thousands of frontline healthcare workers with personal protective equipment and reduce burden in hospitals by providing telemedicine service, we believe such volunteering and initiations from medical students can be an inspiration for all students for future crises.

## INTRODUCTION

There are more than 172 million confirmed cases and more than 3.7 million deaths due to Coronavirus Disease 2019 (COVID-19) pandemic globally.^1^ Nepal is also one of the affected countries with total cases more than five hundred eighty eight thousands and more than seven thousand eight hundred people succumbing their life to the pandemic as on 6^th^ June 2021.^2^ This pandemic shut down the world in a short period of time in a way no one had ever possibly imagined, but the fight against this pandemic proved once again that, human race doesn't give up so easily. Despite all the challenges, the efforts and hard work shown by frontline healthcare workers, security personnel and even the patience shown by the general public by staying at home is highly encouraging.

## ROLE OF MEDICAL STUDENTS

Medical students are future healthcare professionals. In many countries, they are not directly involved in patient care but they could still use their education and knowledge in ways that helped in trying times. In this article, we will be summarizing the efforts by a group of Nepali medical students, including but not limited to the authors, who worked in collaboration with a non-profit non-governmental organization, Nepal Health Corps, during the pandemic.

**1. Awareness Program**

Medical students from Patan Academy of Health Sciences, Nepal conducted awareness programs regarding COVID-19 in schools, colleges, orphanages and old age homes physically before the lockdown. The program included general information about COVID-19, proper ways to wear masks, hand washing techniques and other precautionary methods to prevent COVID-19. During the lockdown, this program was carried out virtually with the help of videos, posters and informative social media posts.

**2. Project PPE**

Owing to the need of Personal Protective Equipment (PPE) among frontline healthcare professionals Nepal Health Corps initiated Project PPE in partnership with different national and international non-profit nongovernmental organizations, most notably Rotary International District 3292 through which we facilitated distribution of around 1000 PPE to the frontlines during April-May 2020.

**3. Project Telemedicine**

As hospitals are places where crowding is unavoidable, there is a high risk of transmission of COVID-19. To minimize patient flow in hospitals, Nepal Health Corps launched Project Telemedicine. Through this initiative, the general public can take free consultation on the telephone from consultants and medical officers from the comfort of their home. This will not only decrease the number of patients visiting hospitals for minor inquiries but also acquaint the people with telemedicine which is a relatively new concept in Nepal.

**4. "Donate Plasma, Help Defeat COVID-19" Campaign**

Convalescent Plasma Therapy may be helpful in decreasing mortality, reducing viral shedding and improving clinical conditions in COVID-19 patients.^3^ Nepal Health Corps initiated and coordinated Donate Plasma, Help Defeat COVID-19 campaign to make convalescent plasma of COVID-19 recovered patients available to critically ill COVID-19 patients. We helped in communication between donor and recipient by convincing potential donors for plasma donation and then made their phone numbers available to recipients when necessary.

We, medical students from Nepal, were not directly involved in taking care of patients, but we had ample time to support our senior healthcare professionals and government in fighting the pandemic as all medical schools were physically closed during the lockdown. We hope that the above-mentioned initiatives will encourage students to contribute in their own ways in future during times of crisis.
